# A Single Nanobelt Transistor for Gas Identification: Using a Gas-Dielectric Strategy

**DOI:** 10.3390/s16060917

**Published:** 2016-06-21

**Authors:** Bin Cai, Zhiqi Song, Yanhong Tong, Qingxin Tang, Talgar Shaymurat, Yichun Liu

**Affiliations:** 1 Key Laboratory of UV Light Emitting Materials and Technology (Northeast Normal University), Ministry of Education, 5268 Renmin Street, Changchun 130024, China; atad2008@126.com (B.C.); songzq530@nenu.edu.cn (Z.S.); tongyh@nenu.edu.cn (Y.T.); 2 Key Laboratory of New Energy and Materials Research, Xinjiang Institute of Engineering, Urumqi 830091, China; talgar.shaymurat@vip.163.com

**Keywords:** gas identification, pristine single nanobelt, field-effect transistor, gas sensor, gas dielectric

## Abstract

Despite tremendous potential and urgent demand in high-response low-cost gas identification, the development of gas identification based on a metal oxide semiconductor nanowire/nanobelt remains limited by fabrication complexity and redundant signals. Researchers have shown a multisensor-array strategy with “one key to one lock” configuration. Here, we describe a new strategy to create high-response room-temperature gas identification by employing gas as dielectric. This enables gas discrimination down to the part per billion (ppb) level only based on one pristine single nanobelt transistor, with the excellent average Mahalanobis distance (MD) as high as 35 at the linear discriminant analysis (LDA) space. The single device realizes the selective recognition function of electronic nose. The effect of the gas dielectric on the response of the multiple field-effect parameters is discussed by the comparative investigation of gas and solid-dielectric devices and the studies on trap density changes in the conductive channel. The current work opens up exciting opportunities for room-temperature gas recognition based on the pristine single device.

## 1. Introduction

One key requirement in the development of gas-analytical sensor systems for environmental monitoring, medical diagnosis, food safety and even homeland security is to reliably recognize a broad range of gases, often in low concentration. Thus far, the realization of such an effective metal oxide semiconductor nanowire/nanobelt gas sensor is still one of the most challenging issues [1,2,3,4]. Most metal oxide semiconductor sensors respond to a number of gases, normally gases of the same group or family, with overlapped electrical signals. By semiconductor composites, surface modification, or/and operation temperature modulation, specific responses to one particular gas with almost zero cross sensitivity, which is referred to as specificity, can be regularly achieved [5,6,7,8,9]. This also provides one available strategy: the multisensor array with a “one key to one lock” configuration, for successful discrimination among a wide variety of gases (Supplementary Materials, Table S1). Each of these sensors or sensing elements is mainly specific to one target analyte. The response electrical signals are recognized as the fingerprints to identify different gases at various concentration levels.

Until now, selective recognition of the multiple gases using the metal oxide semiconductor nanowire/nanobelt is still challenging, due to the complexity of the array fabrication, and the difficulty in distinguishing the potentially redundant signals via various pattern recognition schemes [10,11]. The reported metal oxide semiconductor nanowire/nanobelt sensors for gas identification generally applied a resistor-type array configuration (Table S1), where the current or the conductivity value were applied as the output signals. For example, Moskovits *et al*. reported a promising resistor-type sensor to discriminate three reducing gases (H_2_, CO, and ethylene) by combining the SnO_2_ nanowire sensor array with two ways: temperature gradient (240–285 °C) and nanoparticle modification (pristine, Pd and Ag decoration) [12]. They applied 12 individual nanowire sensing elements (each 4 × 4 array functioned as one sensor element) at four different operating temperatures to obtain the current pattern. Very recently, Liao *et al.* made a remarkable attempt for gas discrimination (CO, C_2_H_5_OH, and O_2_), where a field-effect transistor (FET) array with different metal decoration (pristine, Au, Ag, and Pt) has been employed [10]. In contrast, it is obviously desirable to achieve the recognition capacity and excellent response in one single device at room temperature. The decreased number of sensors can effectively decrease the power consumption and the related computation parts [11,13], and the room-temperature detection can eliminate the heating system for effective work temperature, which has extensively existed in most metal oxide based sensors. Haik *et al.* recently proposed a “specific molecule-modification” strategy on one single Si nanowire to realize the room-temperature recognition of volatile organic compounds [11]. This work, perhaps, is the first example for using a single semiconductor device for selective recognition of the multiple gases. The single-device sensor operated at room temperature has shown several distinct advantages such as simplified fabrication/measurement/data analysis processes, lowered power consumption, improved long-term stability, minimized electrical noise, and enhanced ability of fault tolerance [11,14].

Herein, we present a “gas-dielectric” strategy to design a smart room-temperature operated sensor for gas selective identification based on one single pristine SnO_2_ nanobelt FET. This method avoids the signal noises and the risk of overfitting toward the analyzed data caused by multiple sensing elements, as is the case in the traditional electronic nose approaches. Three poisonous gases, NO_2_, NO, and H_2_S, have been discriminated in extremely low concentration down to part per billion (ppb) levels, through the differentiable multi-parameter response of the FET. Our single nanobelt sensor exhibits quite strong discrimination power with the average Mahalanobis distance (MD) as high as 35 at the Linear Discriminant Analysis (LDA) feature space. The detection concentration is down to 10, 50, 50 ppb towards NO_2_, NO, and H_2_S with extremely high responses at 244%, 360% and 1099%, respectively. The gas dielectric has a decisive effect on the high gas discrimination power and the low detection concentration, and thus opens a new route for the design of a room-temperature operated gas sensor based on one single device.

## 2. Experimental

**Figure 1:**
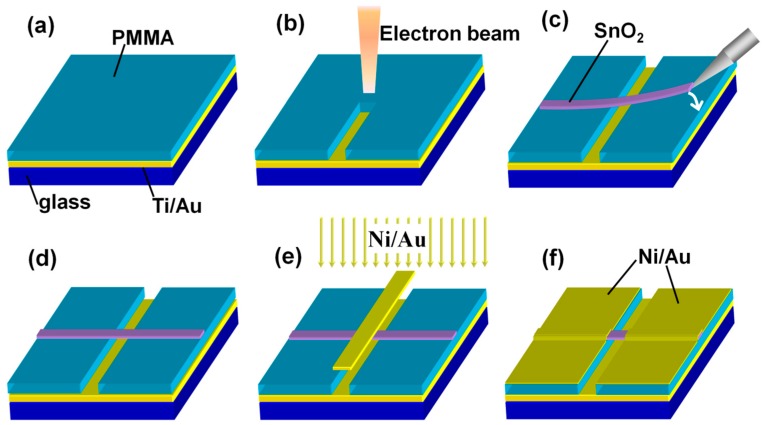
The schematic representation for the fabrication of the gas-dielectric SnO_2_ nanobelt field-effect transistor (FET).

Single crystalline nanowires/nanobelts of SnO_2_ were synthesized by chemical vapor deposition. The individual SnO_2_ nanobelt was transferred by mechanical manipulation with the microprobes of a Cascade 150 M probe station (Beaverton, OR, USA), equipped with an optical microscope, under ambient conditions. The device fabrication process was schematically shown in Figure 1 and was described as followed: (a) the Ti/Au gate electrodes were deposited on an insulated glass substrate, followed by spin-coating a 500-nm polymethyl methacrylate (PMMA) layer (PMMA as the support layer); (b) electron beam lithography was used to remove part of the PMMA and to create a groove with the width ranged from a few micrometers to tens of micrometers; (c,d) a SnO_2_ single crystalline nanobelt was suspended on the groove of the PMMA layer by nanomechanical manipulation; and (e,f) the Ni/Au (40 nm/40 nm) electrodes were deposited by thermal evaporation with a “gold layer“ as a mask. The field-effect and the gas response properties of the devices were recorded at room temperature with a Keithley 4200 SCS (Cleveland, OH, USA) in a homemade stainless steel chamber. Pure dry N_2_, NO_2_, NO and H_2_S were controlled by gas flow meters and were introduced to the testing chamber through the stainless pipes. SEM images were obtained by a Philip XL30 instrument (MicroFEI Philips XL-30 ESEM FEG, Eindhoven, The Netherlands).

## 3. Results and Discussion

### 3.1. Characteristics of the SnO_2_ Field Effect Transistors with Gas Dielectric

**Figure 2:**
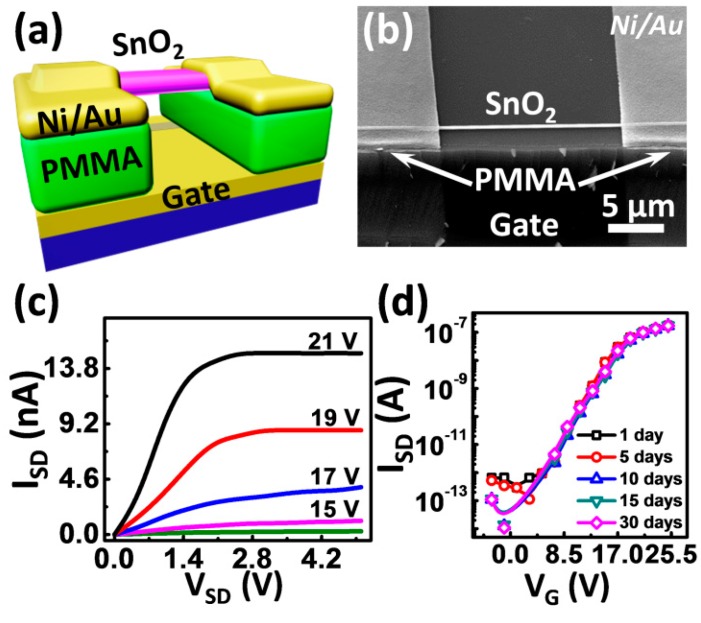
(**a**) the schematic representation of the gas-dielectric SnO_2_ nanobelt FETs; (**b**) the representative SEM image of the device; (**c**,**d**) output and transfer curves of the FET in air at room temperature. All the transfer curves measured in 30 days are overlapped well, which shows the good stability of the device.

Figure 2a shows a schematic image of the sensor. Compared with the resistance type sensor, the three-terminal field-effect configuration can dramatically amplify signals to enhance the response in the sub-threshold regime by the extra gate electrode at room temperature. The gate electrode can regulate the carrier concentration of semiconductors by orders of magnitude. Thus, we can get a certain response even at a lower gas concentration compared with a resistor sensor. The SnO_2_ single crystal nanobelt was selected as a semiconductor sensing layer because of its easy preparation and sensitivity to most of the gaseous species (see Supplementary Materials, Figure S1 for morphology of the nanowires/naonbelts) [15]. The gas dielectric was used to fabricate the FET sensor, so that the gas molecules can adsorb onto the conductive channel of the device directly [16]. The SnO_2_ single crystal nanobelt was mechanically transferred onto a PMMA gap, which was obtained by electron beam lithography (EBL) (see Supplementary Materials, Figure S2 for detailed device fabrication process). The representative SEM image of the device is shown in Figure 2b. For the device measured in the sensing testing, the channel length *L* = 32.9 μm, the width *W* = 230 nm, and the thickness of the dielectric *d* = 500 nm. Figure 2c,d are the output and transfer curves of the device in air at room temperature. The device shows the typical *n*-type output characteristics of the FET with the clear saturation of source-drain current (*I_SD_*) at high source-drain voltage (*V_SD_*). The onset of the curves exhibits the linear characteristics, which confirms a low contact resistance between the SnO_2_ nanobelt and the Ni/Au electrodes [17]. According to the typical transfer curves in Figure 2d, the field-effect mobility (*μ*), on/off ratio, and threshold voltage (*V_T_*) of the device is calculated to be 163.7 cm^2^·V^−1^·s^−1^, 10^6^, and 15.4 V, respectively. We found that the air-dielectric devices always show the excellently reproducible electrical characteristics, regardless of the differences between mobilities, or materials (Figure S3). The air-dielectric SnO_2_ nanobelt device also shows a long-term stability, presented in Figure 2d by the well overlapped curves of multiple measurements in a period of 30 days. The performance is very stable and highly reproducible in both N_2_ and dry air (Figure S4). These results show the ability of the air-dielectric device to provide a platform with a high level of reliability for further sensing measurements.

### 3.2. Gas Sensing Properties

**Figure 3:**
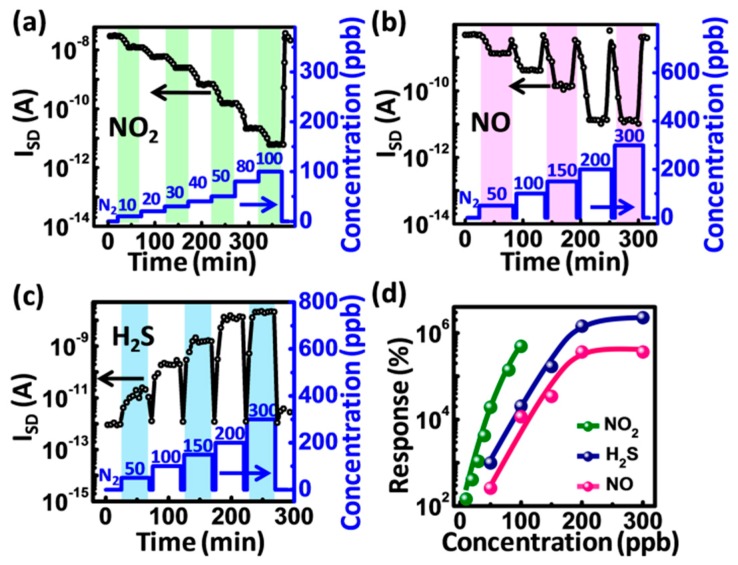
(**a**–**c**) real-time source-drain current (*I_SD_*) response to various concentrations of NO_2_, NO and H_2_S at room temperature. The **blue** line corresponds to the concentration of NO_2_, NO and H_2_S (**right**: *y*-axis); and (**d**) the response of sensors to different analytes in dynamic concentrations.

For NO_2_ semiconductor sensors, for example, the commercialized P/N 706 made by Synkera Technologies, Inc. (Longmont, CO, USA), the interference effect of other NO_x_ is obvious. Here, in order to show the discrimination ability of our sensor to the same kind of component similar gases, we selected NO_2_ and NO as the target gases. In addition, H_2_S was also selected to show the discrimination ability of our sensor to the reducing gases. Figure 3a–c show the real-time *I_SD_* response of a room-temperature operated gas-dielectric device to various concentrations of NO_2_, NO and H_2_S, respectively (test details are available in Supplementary Materials, Figure S5). For the three analytes, the *I_SD_* changes dramatically with the changed gas concentration. It is found that the *I_SD_* decreases or increases by orders of magnitude upon exposure to the target gases, and saturates quickly in every concentration. In Figure 3a, the *I_SD_* shows the stepwise decrease at fixed *V_G_* at room temperature when NO_2_ concentration increases from 0 to 50 ppb with a resolution of 10 ppb. For NO and H_2_S, as shown in Figure 3b,c, the sensor exhibits a stable response and fast recovery behavior in a five-cycle measurement at room temperature in the dark. For NO_2_, the current could return to the baseline rapidly through the use of UV irradiation (at 365 nm in Figure 3a). This observed photodesorption behavior of the adsorbed NO_2_ molecules is in agreement with the previous reports, since the NO_2_ adsorption is irreversible in the dark [18].

The response (*S*) of the device deduced from *I_SD_* is summarized in Figure 3d. The response *S* is calculated according to the following formulas: *S = (I_N_2__ − I_NO_x__)/I_NO_x__* × 100% [19,20,21], where *I_N_2__* and *I_NO_x__*, respectively, are the currents in N_2_ and NO/NO_2_. Since H_2_S acts as a reducing gas and causes the increased current, the response is calculated according to the following formula: *S = (I_H_2_S_ − I_N_2__)/I_N_2__* × 100%. The corresponding response of the sensor to NO_2_, NO and H_2_S, is 244% in 10 ppb, 360% in 50 ppb, and 1099% in 50 ppb, respectively. The permission exposure limits of NO_2_/NO and H_2_S are 53 ppb (annual mean, National Ambient Air Quality Standards) and 10 ppm (10 min ceiling limit, Occupational Safety and Health Administration) [22,23]. Here, the extremely low detection concentration with quite high response and high resolution shows the promising capability of our sensor for the detection of the low-concentration toxic gases (NO_2_, NO, H_2_S) down to ppb levels, which fully meets the requirement of the practical monitoring. To the best of our knowledge, our results are in a class with the lowest detection limit for the metal oxide nanomaterial-based sensor reported so far to identify the three different gases at room temperature [22,24,25,26]. Compared with the nonlinear relationship between gas concentrations and response, the linear dependence of the response at low-concentration NO_2_, NO and H_2_S in Figure 3d also presents the notable advantages of our device for quantitative detection, direct electrical readout, and simplified calibration process and auxiliary circuitry [16].

**Figure 4:**
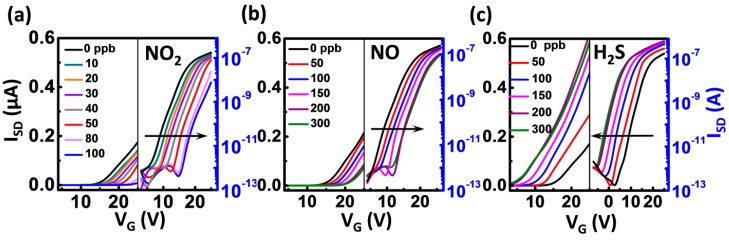
Room-temperature transfer characteristics of the gas-dielectric SnO_2_ nanobelt device to various concentrations of NO_2_, NO and H_2_S. (**a**,**b**) The transfer curves shift monotonically towards the positive direction of gate voltage (V_G_) with the increased NO_2_ and NO concentrations; (**c**) The transfer curves shift monotonically towards the negative direction with the increased H_2_S concentrations.

**Figure 5:**
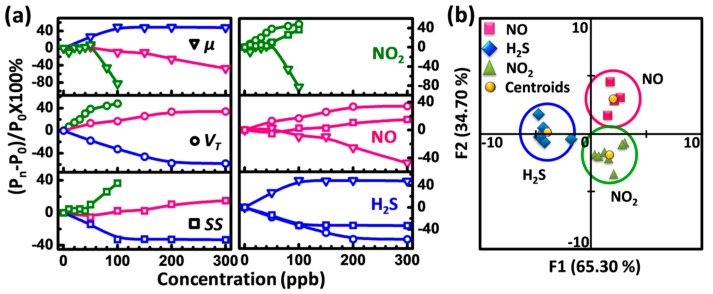
(**a**) percentage variation of a certain parameter at different gas concentrations (**left** panel), and percentage variation of different parameters for a certain gas at different gas concentrations (**right** panel). *μ*: ▽, *V_T_*: ○, *SS*: □; NO_2_: **green**; NO: **pink**, H_2_S: **blue**; (**b**) the results of the **LDA** analysis of the multiple parameters (*μ*, *V_T_*, *SS* and *I_on_*) obtained from the single nanobelt device. The LDA analysis classified and separated the points corresponding to the different gases in dynamic concentration at a 95% confidence level.

Figure 4 is the transfer characteristics of the device measured in dynamic concentrations of analytes. The transfer curves shift monotonically towards the positive direction of *V_G_* with the increased NO_2_ and NO concentrations but shift monotonically to the negative direction with the increased H_2_S concentrations, which is in good agreement with the results in Figure 3. The parameters such as mobility (*μ*), threshold voltage (*V_T_*) and subthreshold swing (*SS*) are extracted from Figure 4, and the percentage variation of the parameters (*P = (P_n_ − P_0_)/P_0_* × 100%, *P_n_* and *P_0_* are referred to as the parameters in the analyte and N_2_, respectively) as a function of concentration is depicted in Figure 5a. Each field-effect parameter presents the obviously different change trend when exposed to different analytes (Figure 5a, left panel), and different parameters also show the dramatically different change trends in the same analyte (Figure 5a, right panel). These promising results indicate that our air-dielectric single nanobelt sensor is capable of discriminating among the three poisonous gases without the need of surface modification and/or temperature modulation.

Based on these dramatically differentiable changes of the field-effect parameters, we used the linear discriminant analysis (LDA), to evaluate the recognition capability of the single device based sensor. LDA is a method for classifying data into categories by constructing a space mapped by vectors formed from linear combinations of predictor variables [27,28]. LDA attempts to choose the linear combination that maximizes the distances separating the locations in space of the transformed predictors. Here, the four parameters (*I_on_*, *μ*, *V_T_* and *SS*) from one single room-temperature operated FET, exposed to the three different toxic gases (NO_2_, NO and H_2_S) at various concentrations, were collected, according to Figure 3a–c and Figure 4. Only 17 data sets were integrated. One data set corresponds to four different parameters (*I_on_*, *μ*, *V_T_* and *SS*), which was obtained from one transfer curve. The aggregate data sets for our single-device gas sensor were processed using the LDA method, and a two-dimensional map was plotted by a software (xlSTAT 2013, Addinsoft, Bordeaux, France), as shown in Figure 5b. A striking feature is that the patterns of three gases are clearly separable and hence can be used to distinguish them. The average MD between analyte-dependent data clusters at the LDA feature space, employed as a quantitative measureament to evaluate the recognition power of the sensor, is ~35. Such performance in our room-temperature operated single-element sensor for gas identification is comparable to the typical array based E-nose composed of 10 sensing elements, combined with temperature gradient and surface modification (MD = 33) [29]. Furthermore, the gas concentrations can be identified by comparing the response electrical parameters in the target analyte within the standard results presented in Figure 5a. Our device with the extremely high discrimination power shows that the combination of the multiple field-effect parameters from one single air-dielectric SnO_2_ nanobelt FET is a promising way to recognize the gases. It realizes the same gas discrimination functions of the traditional electric nose, which is composed of multiple gas sensing elements. It is believed that increasing the number of sensors increases the power consumption, and complicates the device circuitry and the related computation parts [11,30]. The higher the number of the sensing elements, the higher the risk of overfitting toward the analyzed data [11,30]. Thus, the sensor based on one single pristine nanobelt without any surface modification or the fabrication of additional devices also shows the advantages in the simplified device fabrication/measurement/data treatment processes, the minimized noises, the lowered power consumption, and the enhanced integration. Furthermore, the orientation-controlled nanowire using a guiding and stretching strategy to realize the suspended nanowire also provides a reliable way to improve the batch manufacturing of the gas sensor based on the gas-dielectric configuration [31,32,33].

One prerequisite for successful realization of the gas discrimination is to obtain the differentiable signals as pronounced as possible [29,34]. The overlapping response signals make the discrimination difficult and even failed. In order to provide a sufficient discrimination power, some of the previously reported metal oxide semiconductor nanowire/nanobelt E-noses achieved the diversity in the gas response based on the differences between devices and/or the differences between measurement conditions (Table S1), for example, decorated metal nanoparticle species, temperature gradient, nanowire diameter, nanowire density, etc. In our experiments, we employed the multiple field-effect parameters of one single device to realize the diversity of the gas response. Toris et al. compared the parameters of an organic thin film FET in pure N_2_, O_2_, and the mixture of the H_2_O and N_2_ atmosphere [35]. In principle, this provides an attractive way to realize the new generation of gas identification based on a single field-effect device. Until now, however, little progress on the multi-parameter model has been made [11,36]. One possible reason is the commonly used solid dielectric in FETs. We comparatively investigated the gas response properties of the solid-dielectric SnO_2_ nanobelt device (Figure S6). The experiment results show that all field-effect parameters in the solid-dielectric device were unchanged in H_2_S (50–300 ppb). When exposure upon to NO_2_ and NO, *SS* and *μ* of the solid-dielectric device present the weak response. Therefore, the gas dielectric possibly is favorable for the improved gas discrimination capability only based on an individual pristine nanobelt. Our previous results have shown that the gas adsorption in the conductive channel of FETs have the far larger influence than on the upper and side surfaces of the semiconductor nanowire [16]. The conductive channel of the traditional solid dielectric FET is capped by the semiconductor layer and the solid dielectric. In contrast, the gas dielectric makes the conductive channel exposed to the detected gas. The gas dielectric facilitates the direct interaction between the gas molecules and the conductive channel [19], and further amplifies the sensing signal with the modulation of gate voltage, which is responsible for the low limit of detection (LOD) and the dramatically differentiable parameter changes. Compared with other single SnO_2_ nanowire/nanobelt sensors, although the sensitivity, LOD and other sensing parameters are influenced by the synthesis condition, the size of nanowire/nanobelt, the working voltage or operation temperature [37,38,39,40,41], the gas dielectric still plays an outstanding role and significantly enhances the sensing performances of SnO_2_ nanobelts.

**Figure 6:**
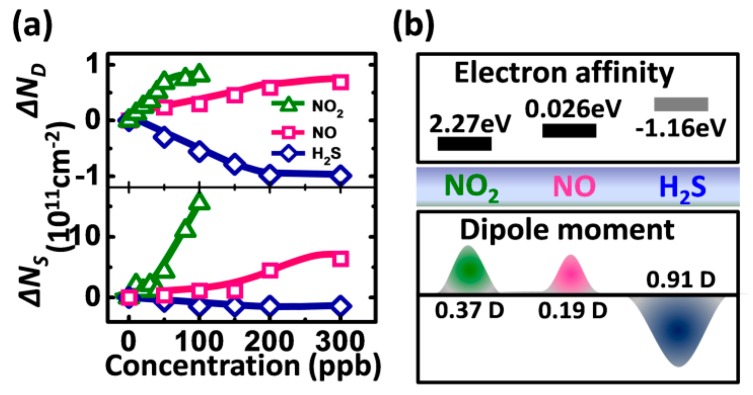
(**a**) changes of deep trap density (*ΔN_D_*) and shallow trap density (*ΔN_S_*) at semiconductor/dielectric interface as a function of NO_2_ (**green** triangle), NO (**pink** square) and H_2_S (**blue** rhombic) in dynamic concentrations; and (**b**) electron affinity and dipole moment of NO_2_, NO and H_2_S.

It has been addressed that, in FETs, the deep trap density in the conductive channel could shift *V_T_*, and the shallow trap density in the conductive channel could modulate *μ* and *SS* [16,42,43]. In order to deeply understand the effect of the gas molecules adsorbed in the exposed conductive channel on the field-effect parameters, we further investigate the changes of the deep and shallow trap densities before and after gas adsorption in our gas-dielectric device. In FETs, the changed deep trap density *ΔN_D_* and the changed shallow trap density *ΔN_S_* can be respectively approximately calculated by the following equations [16,42,43],

(1) $$\Delta N_{D}=\Delta V_{T}C_{i}\;/q$$

(2) $$\Delta N_{S}=\frac{\Delta SS\times C_{i}}{KTln10}$$

where *K* is Boltzmann’s constant, *T* is absolute temperature, $C_{i}$ is the calculated capacitance per unit area of the insulator, and *q* is electron charge. According to the results of Figure 5a, the dependence of the changed trap density (*ΔN_S_* and *ΔN_D_*) on the analyte concentration in our gas-dielectric device is shown in Figure 6a using Equations (1) and (2). *ΔN_D_* and *ΔN_S_* present the different change trends upon exposure to different gases. The slopes of *ΔN_D_* and *ΔN_S_* in the linear regions are different and the abrupt turning point occurs at different concentrations for different gases. We suppose that such changes of *ΔN_D_* and *ΔN_S_* are possibly related to electron affinity and dipole moment of the adsorbed molecules. Figure 6b shows the electron affinity and dipole moment of the three gases [44,45,46,47,48,49]. It has been reported that an analyte with higher electron affinity has stronger binding to the electrons, which works as deep traps [50], and the polar molecules adsorbed in the channel can form bound states with carriers that are temporarily localized on shallow traps [51]. Those oxidizing gas molecules first preferentially adsorb to the defect sites in conductive channels as the deep trap centers, and bound the electrons to increase the density of deep traps [52]. In addition, then the shallow trap centers are formed with the increased gas concentration. As a result, as shown in Figure 6a, *ΔN_D_* in NO_2_ and NO first changes linearly with the concentration and then tends to saturation from the abrupt turning point. However, *ΔN_S_* almost remains unchanged at the low concentration, and then begins to increase or decrease. The saturated |*ΔN_D_*| in NO_2_ is ~10^11^ cm^−2^, which is almost the same as in NO and H_2_S. This further suggests that the deep defect sites where the gas molecules can adsorb in the channel are limited, resulting in the saturation of the deep traps. The dipole moment of the polar molecules changes the dipole-charge interaction, and hence affects the trap states and the carrier transport [51]. For oxidizing gases such as NO_2_ and NO, the deep and shallow trap states capture the electrons that are initially available for the carrier transport. Higher positive electron affinity and dipole moment, more electrons are bound in our gas-dielectric device, resulting in higher slopes of *ΔN_D_* and *ΔN_S_* in the linear regions for NO_2_ and NO. For the reducing gas with the negative electron affinity, the molecules adsorbed in the channel donate the electrons that effectively fill the deep and shallow defect traps, resulting in the decreased *ΔN_D_* and *ΔN_S_* with the concentration.

## 4. Conclusions

The room-temperature operated gas identification has been fabricated based on one individual pristine nanobelt FET with a “gas-dielectric” strategy. The gas dielectric provides the ability to perfectly distinguish among three analytes (NO_2_, NO, and H_2_S), with detect concentrations down to ppb levels, by the differentiable multi-parameter responses among them. The pristine single nanobelt sensor exhibits excellent discrimination power with the average MD as high as 35, and an extremely high response. The response towards 10 ppb NO_2_, 50 ppb NO, and 50 ppb H_2_S can reach up to 244%, 360%, and 1099%, respectively. Additionally, the gas concentrations can be identified by contrasting the response signals with the standard data. These outstanding results probably demonstrate the simplest processes in fabrication, measurement, and data analysis from gas identification, and open up a new strategy for room-temperature gas recognition based on the single device. The electron affinity and dipole moment of the test gas are possibly responsible for the adsorption process in the exposed conductive channel, resulting in the differentiable multi-parameter response.

## Supplementary Materials

The following are available online at http://www.mdpi.com/1424-8220/16/6/917/s1; Figure S1: SEM images of SnO_2_ nanowires/nanobelts; Figure S2: Schematic representations of the device fabrication process; Figure S3: The multimeasured transfer curves of the gas-dielectric nanowire FETs with different mobilities and semiconductor materials; Figure S4: Transfer curves of the gas-dielectric SnO_2_ nanobelt based FET tested in dry air and N_2_; Figure S5: Schematic images of the experimental setup used for gas sensing; Figure S6: Response to three analytes (NO_2_, NO and H_2_S) in a solid-dielectric device; and Table S1: Strategies and models for the reported metal oxide semiconductor (MOS) based E-noses.
