# Supplementary material for: A Single Nanobelt Transistor for Gas Identification: Using a Gas-Dielectric Strategy
Source: Sensors (Basel). 2016 Jun 21;16(6):917. doi: 10.3390/s16060917 (PMC4934343; doi:10.3390/s16060917)
Supplement: Supplementary file 1 [file sensors-16-00917-s001.pdf]

Supplementary Materials: A Single Nanobelt Transistor for Gas Identification: Using a Gas-Dielectric Strategy

Bin Cai, Zhiqi Song, Yanhong Tong, Qingxin Tang, Talgar Shaymurat and Yichun Liu

1. Strategies and models for the reported MOS based E-noses.
2. SEM images of SnO_2 nanowires/nanobelts.
3. Schematic diagrams of the device fabrication process.
4. Multiple measurement results of gas-dielectric devices.
5. Testing process for gas sensing.
6. Response to three analytes (NO_2, NO and H_2S) in solid-dielectric device.

1. Table S1. Strategies and models for the reported metal oxide semiconductor (MOS) based E-noses.

Material	Type	Operating Temperature (°C)	Surface Modification	Target Species	Data Evaluation Modeling	Ref.
SnO_2 nanowire array	Resistor	240–285	Pristine<br>Ag<br>Pd	H_2<br>CO<br>C_2H_4	LDA [a]	[1]
SnO_2 nanobelt with changed diameter	Resistor	290	Pd with changed density	Toluene (1–30 ppm)<br>Ethanol (1–30 ppm)<br>2–Propanol (1–30 ppm)<br>CO (1–30 ppm)	LDA	[2]
SnO_2 nanowire<br>In_2O_3 nanowire<br>SnO_2:Ni nanowire<br>TiO_2 nanowire	Resistor	350	–	H_2<br>CO	Radial plots of the response signals	[3]
SnO_2 nanowire array with changed density	Resistor	247–327	–	2–Propanol (0.5–50 ppm)<br>Ethanol (0.5–50 ppm)<br>CO (0.5–10 ppm)	LDA	[4]
SnO_2 nanowire array	Resistor	192–373	–	Acetone (20–80 ppm)<br>Ethanol (20–80 ppm)<br>MEK [b] (20–80 ppm)	LDA	[5]
CNT-SnO_2 film	Resistor	250–300	–	Ethanol (100–1000 ppm)<br>Methanol (100–1000 ppm)	-	[6]
In_2O_3 nanowire<br>ZnO nanowire<br>SnO_2 nanowire<br>SWNT array	Resistor	25<br>200	–	H_2 (500–2000 ppm)<br>Ethanol (50–200 ppm)<br>NO_2 (0.1–1 ppm)	PCA [c]	[7]
In_2O_3:Mg nanowire	FET	RT	Pristine<br>Au<br>Ag<br>Pt	CO (0.5–100 ppm)<br>Ethanol (100 ppm)<br>H_2 (100 ppm)	-	[8]
Single SnO_2 nanowire	FET	RT	–	NO_2 (10–100 ppb)<br>NO (50–300 ppb)<br>H_2S (50–300 ppb)	LDA	Our work

[a] LDA: Linear Discriminant Analysis; [b] MEK: Methylethylketone; [c] PCA: Principal Component Analysis.

2. SEM Images of SnO_2 Nanowires/Nanobelts

Single-crystal nanowires/nanobelts of SnO_2 were synthesized by vapor transport as previously reported [9]. The nanowires/nanobelts have the regular shape and the smooth surface with diameters of 100–800 nm and lengths of tens of micrometers.

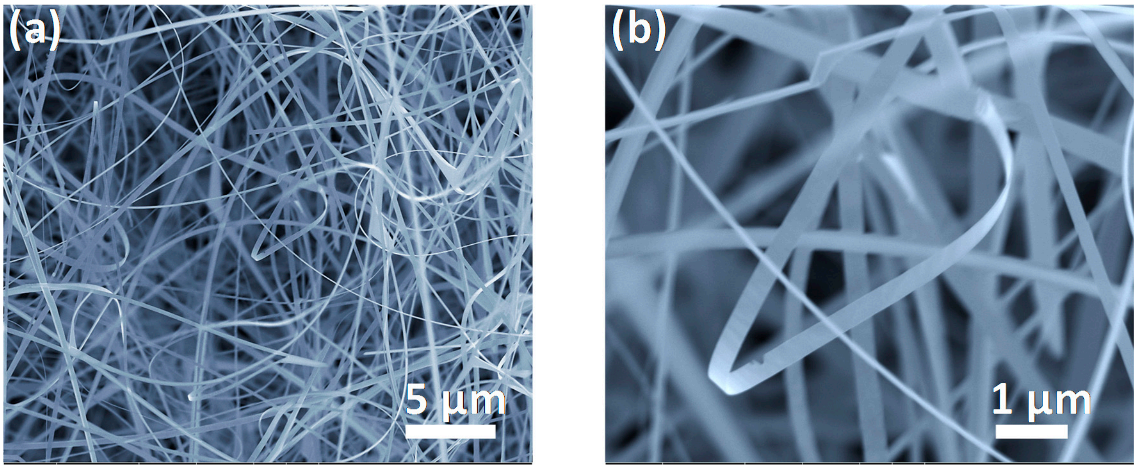

Figure S1. SEM images of SnO_2 nanowires/nanobelts.

3. Fabrication Process of SnO_2 Nanobelt FET with Gas Dielectric

The device fabrication process is shown in Figure S2. (a) The patterned Ti/Au gate electrodes were deposited on the insulated glass substrate by ultraviolet lithography. The polymethyl methacrylate (PMMA) layer was spin-coated onto the substrate as dielectric. The thickness of the PMMA layer is 500 nm; (b) Electron Beam Lithography was used to remove part of the PMMA and to create a groove with the width ranged from a few micrometers to tens of micrometers; (c) A single-crystal nanobelt of SnO_2 was suspended on the groove of the PMMA layer by nanomechanical manipulation; (d) The gold films were placed by a " stamping gold layer" technique [10], which serves as the mask for source-drain electrode deposition; (e) The Ni/Au (40 nm/40 nm) electrodes were deposited by thermal evaporation; (f) The adhered gold films were removed by mechanical probe.

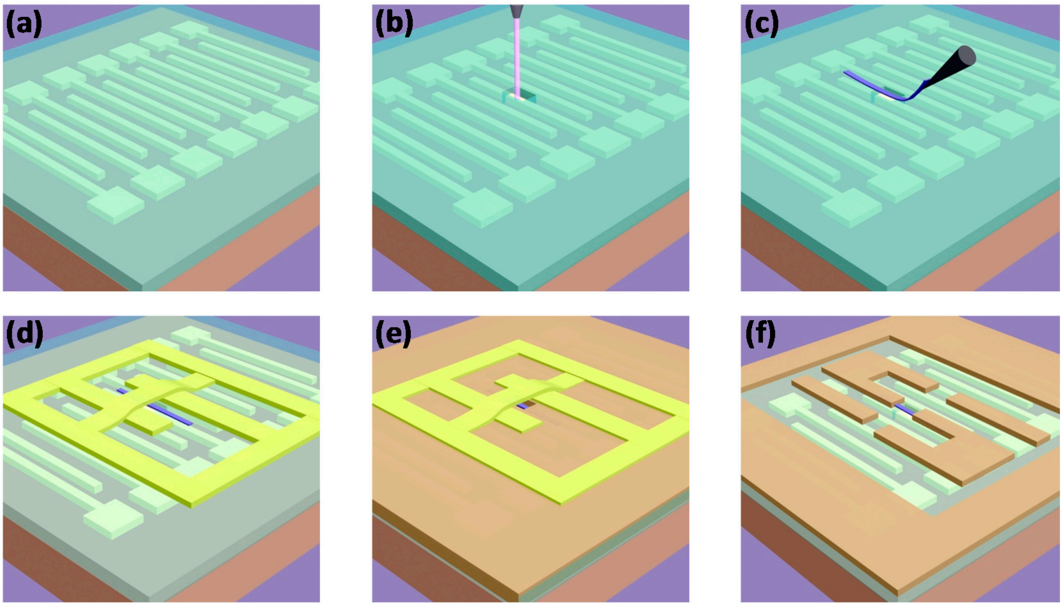

Figure S2. Schematic representations of the device fabrication process.

4. Multiple Measurement Results of Gas-Dielectric Devices

The comparative results in Figure S3 show that both SnO_2 and CuPc nanowire FETs with gas dielectric present the excellently reproducible electrical characteristics. For the same semiconductor material, both low and high-mobility devices present the good repeatable electrical characteristics.

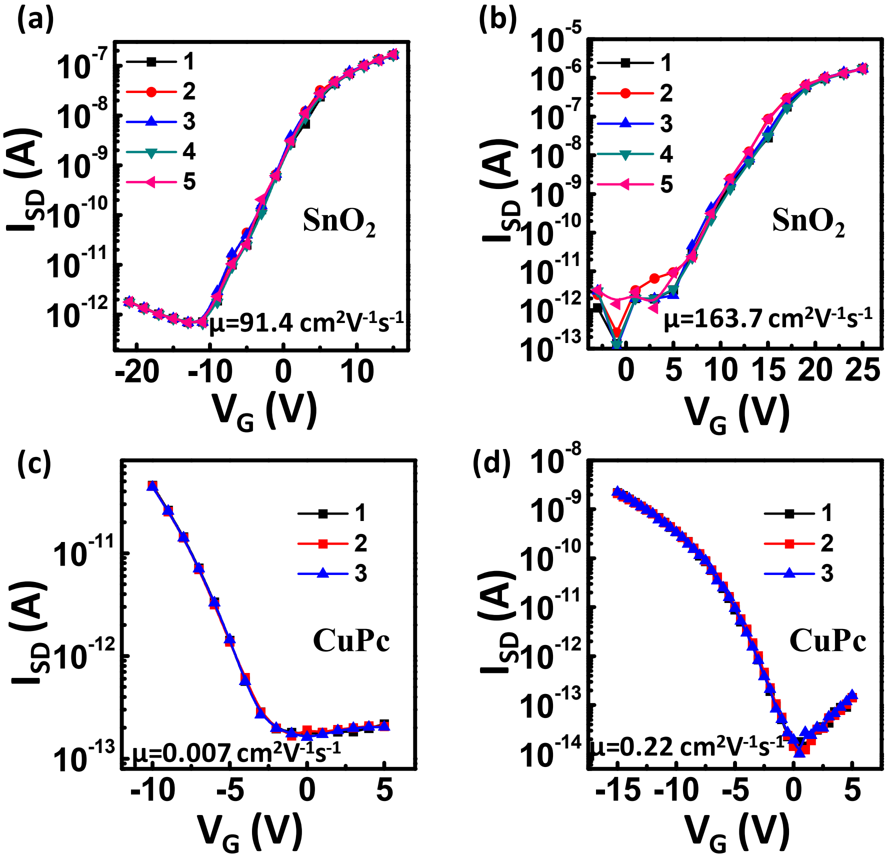

Figure S3. The multimeasured transfer curves of the gas-dielectric nanowire FETs with different mobilities and semiconductor materials: (**a**) SnO_2 with mobility at 91.4 $cm^2 \cdot V^{-1} \cdot s^{-1}$; (**b**) SnO_2 with mobility at 163.7 $cm^2 \cdot V^{-1} \cdot s^{-1}$; (**c**) CuPc with mobility at 0.007 $cm^2 \cdot V^{-1} \cdot s^{-1}$; (**d**) CuPc with mobility at 0.22 $cm^2 \cdot V^{-1} \cdot s^{-1}$.

As shown in Figures S4, the electrical characteristic of the SnO_2 nanobelt FET with gas dielectric was measured in dry air, and then in N_2. The measurements in N_2 were carried out after the N_2 stream was introduced into the chamber for 1 h and 3 h, respectively. These measured results show that the device performance is highly repeatable both in dry air and N_2.

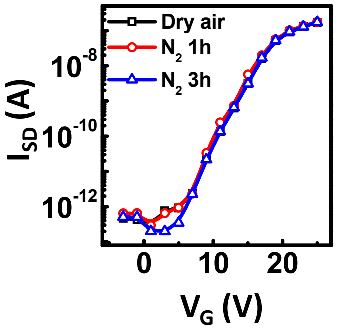

Figure S4. Transfer curves of the gas-dielectric SnO_2 nanobelt based FET tested in dry air and N_2.

5. Testing Process for Gas Sensing

Figure S5 shows a schematic representation of the experimental setup for gas detection. The device was placed into the stainless testing chamber and its leading wires were connected to a Keithley 4200-SCS station, for electrical characterization. The testing gas was introduced to the chamber through the stainless pipes. Prior to the testing, pure dry N_2 was introduced into the chamber for 2 h, so as to purge the testing chamber of undesired residual gases. The electrical characteristics of the FET nanosensor were first measured in the N_2 for 2 h to confirm the stability of devices. Subsequently, the testing gas was introduced to the chamber and diluted by N_2. The stream of N_2 and the testing gas was introduced under the controlled flow rate, by Mass Flow Controllers (MFC CS200A). The total gas flow rate was kept at 500 sccm. At the end of the testing cycle, the testing gas was collected by an alkaline solution.

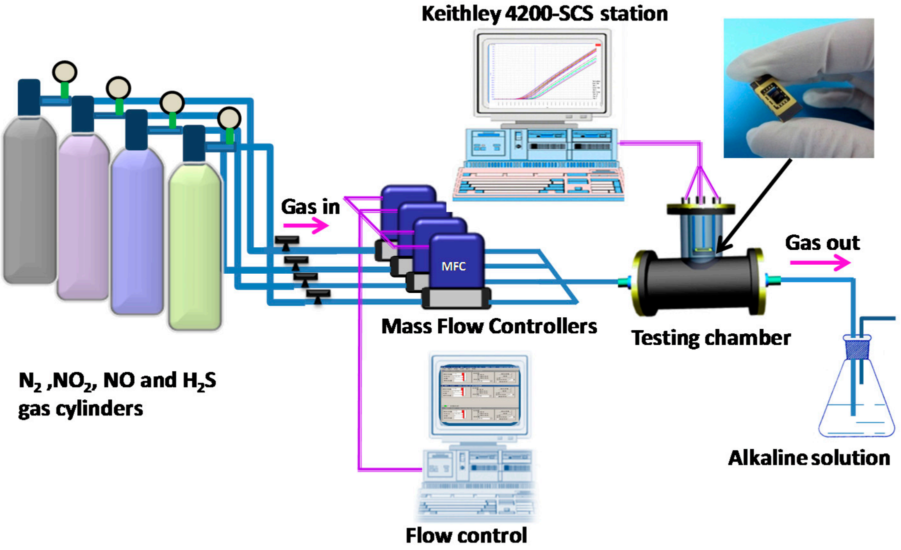

Figure S5. Schematic images of the experimental setup used for gas sensing.

6. Response to Three Analytes (NO_2, NO and H_2S) in a Solid-Dielectric Device

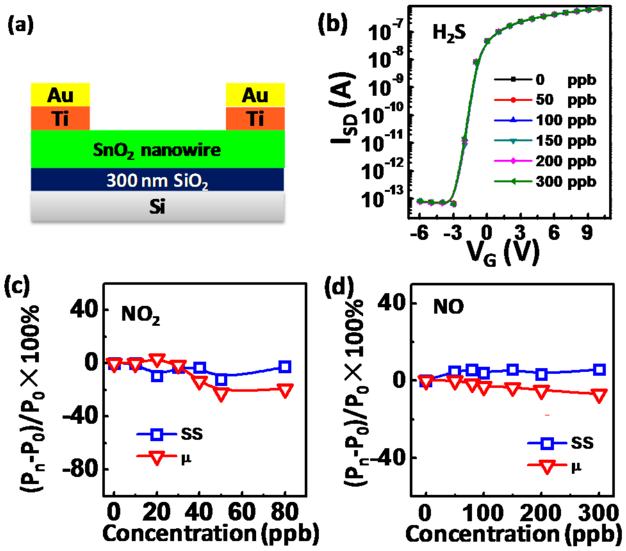

Figure S6. (**a**) Schematic image of a solid-dielectric nanobelt device; (**b**) Transfer curves of the solid-dielectric device to various concentrations of H_2S. The well overlapped curves show that the solid-dielectric device does not respond to H_2S; (**c**, **d**) Parameter percentage variation at different NO_2 and NO concentrations in solid-dielectric device. All electrical measurements were carried out at room temperature.

References

1. Baik, J.M.; Zielke, M.; Kim, M.H.; Turner, K.L.; Wodtke, A.M.; Moskovits, M. Tin-oxide-nanowire-based electronic nose using heterogeneous catalysis as a functionalization strategy. *ACS Nano* **2010**, *4*, 3117–3122.
2. Sysoev, V.V.; Strelcov, E.; Sommer, M.; Bruns, M.; Kiselev, I.; Habicht, W.; Kar, S.; Gregoratti, L.; Kiskinova, M.; Kolmakov, A. Single-nanobelt electronic nose: Engineering and tests of the simplest analytical element. *ACS Nano* **2010**, *4*, 4487–4494.
3. Sysoev, V.V.; Button, B.K.; Wepsiec, K.; Dmitriev, S.; Kolmakov, A. Toward the nanoscopic "electronic nose": Hydrogen *vs* carbon monoxide discrimination with an array of individual metal oxide nano-and mesowire sensors. *Nano Lett*. **2006**, *6*, 1584–1588.
4. Sysoev, W.; Goschnick, J.; Schneider, T.; Strelcov, E.; Kolmakov, A. A gradient microarray electronic nose based on percolating SnO_2 nanowire sensing elements. *Nano Lett*. **2007**, *7*, 3182–3188.
5. Dattoli, E.N.; Davydov, A.V.; Benkstein, K.D. Tin oxide nanowire sensor with integrated temperature and gate control for multi-gas recognition. *Nanoscale* **2012**, *4*, 1760–1769.
6. Wongchoosuk, C.; Wisitsoraat, A.; Tuantranont, A.; Kerdcharoen, T. Portable electronic nose based on carbon nanotube-SnO_2 gas sensors and its application for detection of methanol contamination in whiskeys. *Sens. Actuators B Chem.* **2010**, *147*, 392–399.
7. Chen, P.C.; Ishikawa, F.N.; Chang, H.K.; Ryu, K.; Zhou, C. A nanoelectronic nose: A hybrid nanowire/carbon nanotube sensor array with integrated micromachined hotplates for sensitive gas discrimination. *Nanotechnology* **2009**, *20*, 125503.
8. Zou, X.; Wang, J.; Liu, X.; Wang, C.; Jiang, Y.; Wang, Y.; Xiao, X.; Ho, J.C.; Li, J.; Jiang, C.; *et al*. Rational design of sub-parts per million specific gas sensors array based on metal nanoparticles decorated nanowire enhancement-mode transistors. *Nano Lett*. **2013**, *13*, 3287–3292.
9. Wan, Q.; Dattoli, E.N.; Lu, W. Appl. Transparent metallic Sb-doped SnO_2 nanowires. *Phys. Lett.* **2007**, *90*, 222107.
10. Tang, Q.; Tong, Y.; Li, H.; Ji, Z.; Li, L.; Hu, W.; Liu, Y.; Zhu, D. High-Performance Air-Stable Bipolar Field-Effect Transistors of Organic Single-Crystalline Ribbons with an Air-Gap Dielectric. *Adv. Mater.* **2008**, *20*, 1511–1515.
